# Dual echo gradient echo imaging for simultaneous thermal mapping in cortical bone and soft tissue

**DOI:** 10.1186/2050-5736-3-S1-O47

**Published:** 2015-06-30

**Authors:** Elizabeth Ramsay, Charles Mougenot, Mohammad Kazem, Theodore W Laetsch, Rajiv Chopra

**Affiliations:** 1 Sunnybrook Research Institute, Toronto, Canada; 2 Philips Healthcare Canada, Toronto, Canada; 3 University of Texas Southwestern Medical Center, Dallas, Texas, United States

## Background/introduction

MRI-guided high-intensity focused ultrasound (MR-HIFU) therapy can relieve pain associated with metastatic and benign bone tumours in patients who fail to respond to conventional radiation therapy. However, since existing MR-thermometry techniques do not provide temperature information within the bone, HIFU exposures in bone are currently monitored using temperature changes in adjacent soft tissues. In this study, a standard dual echo spoiled gradient echo (SPGR) sequence is proposed to monitor thermal effects in both bone and soft tissue simultaneously. Magnitude signal changes at the shorter TE (~1ms) reflect thermal changes in cortical bone, while phase changes at the longer TE (~10ms) allow conventional PRF thermometry in surrounding tissues.

## Methods

**Figure 1:**
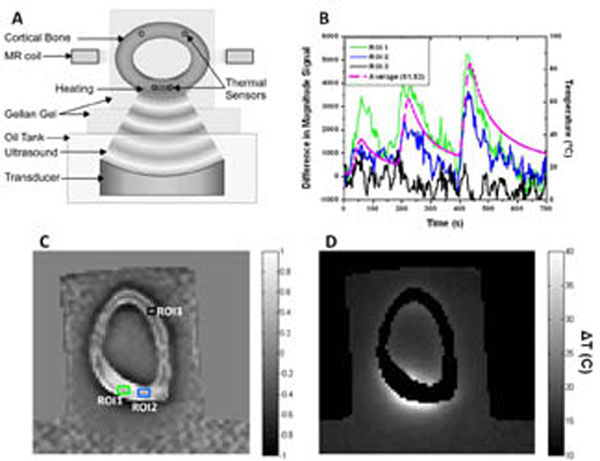
(A) Schematic of HIFU heating experiment. (B) The left axis shows the magnitude signal change observed in the ROIs shown in C during heating. The ROIs are centered on the sensor positions. The right axis shows the average temperature of sensors 1 and 2. (C) Correlation coefficient map of the average temperature of sensors 1 and 2 with signal change derived from TE1 magnitude images. The correlation is positive in the heated area within the bone, negative in the gel outside the bone, and negligible for areas of the bone distant from the treated region. (D) PRF temperature change in the gel surrounding the bone, derived from the TE2 phase images.

As shown in Fig 1A, *ex vivo* cortical beef bones were stripped of marrow and connective tissue, embedded in gel, and sonicated using a Sonalleve V2 HIFU system (Philips Healthcare). A proximal region of the bone was exposed to ultrasound for periods of 30 seconds at powers of 20-60 W, while a dual echo SPGR (echo times TE1 = 1 ms, TE2 = ~ 10 ms) sequence was run repeatedly using a 3T Achieva MRI system (Philips). Bone temperature was measured as a function of time using fiber-optic temperature sensors (Neoptix) inserted in pre-drilled holes in the bone, two in the heated region and two distant from the region of heating. The correlation of the temperature with magnitude and phase images at the two echo times was examined.

## Results and conclusions

As shown in the Figure, local cortical bone temperature changes were well-correlated temporally (1B) and spatially (1C) with changes in signal magnitude at short (~1ms) echo times, while temperature in the gel could be measured via changes in the voxel phase at long (10ms) echo times (1D). These results demonstrate a simple method for monitoring thermal changes simultaneously in cortical bone and soft tissue using a dual echo gradient echo sequence. The technique can be easily translated onto existing MR imaging systems thus improving the safety of MR HIFU treatments.

